# Technology-Enabled Collaborative Care for Type-2 Diabetes and Mental Health (TECC-D): Findings From a Mixed Methods Feasibility Trial of a Responsive Co-Designed Virtual Health Coaching Intervention

**DOI:** 10.5334/ijic.7608

**Published:** 2024-02-16

**Authors:** Diana Sherifali, Carly Whitmore, Farooq Naeem, Osnat C. Melamed, Rosa Dragonetti, Erika Kouzoukas, Jennifer Marttila, Frank Tang, Elise Tanzini, Seeta Ramdass, Peter Selby

**Affiliations:** 1 School of Nursing, McMaster University, Hamilton ON Canada; 2 Population Health Research Institute, Hamilton ON Canada; 3 Nicotine Dependence Service, Centre for Addiction and Mental Health, Toronto ON Canada; 4 Campbell Family Mental Health Research Institute, Centre for Addiction and Mental Health, Toronto ON Canada; 5 Department of Family and Community Medicine, University of Toronto, Toronto ON Canada; 6 Diabetes Action Canada, Toronto ON Canada; 7 Office of Social Accountability and Community Engagement, McGill University, Montreal QC Canada; 8 Department of Psychiatry, University of Toronto, Toronto ON Canada; 9 Dalla Lana School of Public Health, University of Toronto, Toronto ON Canada

**Keywords:** Type 2 diabetes, mental health, integrated care, virtual care, health coaching

## Abstract

**Introduction:**

Type-2 diabetes (T2D) is a complex chronic condition associated with a lower quality of life due to disease specific distress. While there is growing support for personalized diabetes programs, care for mental health challenges is often fragmented and limited by access to psychiatry, and integration of care. The use of communication technology to improve team based collaborative care to bridge these gaps is promising but untested.

**Methods:**

We conducted an explanatory sequential mixed methods study to assess the feasibility and acceptability of the co-designed Technology-Enabled Collaborative Care for Diabetes and Mental Health (TECC-D) program. Participants included adults aged ≥18 years who had a clinical diagnosis of T2D, and self-reported mental health concerns.

**Results:**

31 participants completed the 8-week virtual TECC-D program. Findings indicate that the program is feasible and acceptable and indicate that there is a role for virtual diabetes and mental health care.

**Discussion:**

The TECC-D program, designed through an iterative co-design process and supported by innovative, responsive adaptations led to good uptake and satisfaction.

**Conclusion:**

The TECC-D model is a feasible and scalable care solution that empowers individuals living with T2D and mental health concerns to take an active role in their care.

## Introduction

Type 2 diabetes (T2D) is a complex condition that contributes to further health complications, including mental health disorders that impact quality of life. Among those adults living with T2D, the prevalence of co-occurring mental illness such as depression can be as high as 34% for women and 23% for men – twofold higher than the general population [1,2]. However, diabetes distress, the psychological consequences of living and managing diabetes, is even more common, with an overall presence of 36% of adults with T2D living with the negative feelings, anger, fear, guilt, frustration, and shame associated with this chronic condition [3].

There is a growing need for personalized diabetes programs including self-management education, support, and access to care providers, including diabetes and mental health expertise [4]. Given the high prevalence of co-morbid mental health concerns in those living with T2D, it reveals the need for integrated mental and diabetes care at multiple levels; further, it showcases the broad range of skills, experiences, and knowledge required to adequately and fulsomely provide treatment, education, and ongoing support [5,6]. For individuals living with diabetes and co-occurring mental health challenges including anxiety, depression, or addiction, care typically is delivered not only across different care providers, but often in different care settings with little to no communication between these providers or settings [7,8,9]. This problem is often referred to as siloed or fragmented care, and is particularly important when considering the high rates of mental health concerns that are present in people with T2D [10,11]. Furthermore, those living with T2D and untreated mental health concerns, issues, and challenges tend to have poor diabetes and overall health outcomes [12,13].

The COVID-19 pandemic brough to light a unique series of challenges where individuals were living with limited access to interprofessional diabetes education, care, and follow-up, and also living with limited support for comorbidities such as distress and depression which may have been exacerbated during the pandemic [14,15,16]. In parallel to these challenges, we had the additional challenge of the rapid digitalization of care that occurred. This brought challenges specific to existing disparities, including for those living with digital poverty or decreased digital literacy coupled with intentional efforts to prevent exacerbation of these inequities [15,16]. To address similar challenges, we had previously tested a technology-enabled collaborative care model for youth with early psychosis (TECC-Y) [17]. In this study, it was found that a high-intensity of virtual touch-points (i.e., one-on-one visits for up to 60 minutes for up to 12 weeks) had a positive impact on self-perceived benefit of health behaviour change compared to those who received fewer and less intense support [17].

Despite recent attempts to circumvent such barriers through the virtual delivery of diabetes programs, few have demonstrated effectiveness and scalability due to: a) limited patient access to technology; b) limited patient-involvement in the development, impacting patient acceptability and adherence; and c) limited integration of care, thus further fragmenting care and support [14]. Such programs are costly and resource intensive, further increasing the impact of existing geographical, social, cultural, and financial barriers, creating greater health burdens on underserviced and underrepresented populations [14,15,16].

Building on the findings of the TECC-Y study, the objectives of this feasibility study included: 1) Assessing the feasibility of the Technology-Enabled Collaborative Care for Diabetes and Mental Health (TECC-D) program including assessing recruitment strategies and participant engagement; and 2) Understanding participant, health coach, and virtual care team experience and satisfaction with the program.

## Methods

### Design

This feasibility study used an explanatory sequential mixed methods design to evaluate the feasibility of this co-designed program. This included the collection of quantitative data with subsequent qualitative data collection to augment understanding of study objectives.

#### Quantitative design and sampling

A single-group feasibility study was designed to gather preliminary understanding of recruitment and engagement data as well as to explore the degree of variability, over time, in baseline data collected. To allow for common challenges in the implementation of this intervention and to reflect the expected diversity of participant demographics, clinical presentation, and comorbid health conditions, we set a sample size of 30 participants. Participants were recruited from the Smoking Treatment for Ontario Patients (STOP) program [18]. STOP is a free of charge, Ontario-wide initiative delivered through community health care organizations for smoking cessation treatment and counselling support to people who want to reduce or quit their tobacco use. Participants who had consented for future research within the STOP database and had a self-reported diagnosis of diabetes were contacted by a research assistant. This convenience sample of participants included individuals who currently or previously smoked cigarettes. Participants were eligible for this feasibility study if they lived in Ontario, were aged 18 years and older, had a self-reported a diagnosis of T2D for at least one year, self-reported living with mental health challenges, had access to a telephone or internet through a computer or mobile phone, and were able to understand, write, and read in English.

#### Qualitative design and sampling

A qualitative descriptive study design was used to understand the experience and acceptability of the TECC-D intervention. This design involves a close, comprehensive summary of experience data using the language of participants to provide rich, in-depth understanding. All participants were invited to participate in an interview following the completion of the intervention (please see Supplemental File 1 for the participant interview guide). Each member of the virtual care team was also invited to complete an interview once the study was completed. No further eligibility criteria were applied, and for their involvement, participants who completed an interview were provided a $25 gift card. Interviews were designed and led by a PhD trained qualitative health researcher with clinical training in psychiatric and mental health care (CW). The interviewer was unknown to the participants prior to the interview.

#### Co-design

Recognizing the need to design and adapt an intervention that addresses the complexity of living with both diabetes and mental health concerns, we used a co-design approach with health care professionals (physicians, nurses, diabetes and mental health specialists), researchers, and three patient partners. Patient partners were people with lived experience. They represented diverse voices, including those from structurally marginalized and racialized populations, those who did not speak English as a first language, and who live with different accessibility needs.

Integrated care models are complex interventions that seek to integrate services within and across organizational and professional boundaries. These models themselves are complex not only because of their use of governance, systems, and technology to achieve these aims, but because of the often complex nature of the individuals they serve [19]. Reflective of a deliberate shift toward people-driven care [20], rather than merely people-centred care, co-design centers the expressed needs of those living with the condition in the design process. Building on existing team assets, this co-design work included the re-design of the previously applied TECC model [17,21], the use of widely available technology, the application of health coaching [22,23,24], and the use of the STOP database [25]. Further, and to support future scale-up and further testing of this program, widely used omni-channel technology was used to both deliver the program and support data collection. This included the use of Webex Teams and REDCap. Co-design drove initial decision-making about the study design, questionnaires used, and intervention processes. Throughout the course of the study, weekly design meetings were held with a sub-team of study investigators and providers to iteratively co-design and adapt the intervention on an ongoing basis. This was responsive to participant needs, coach and virtual care team observations, and operated as a quasi-learning health system to make agile adjustments and adaptations to the model and its delivery in real time.

### Intervention

#### TECC-D Program

The TECC-D program was an eight-week virtual, collaborative care intervention that paired participants with a health coach to help create and achieve goals, provide education and support, and explore health and wellness. The health coach, a Certified Diabetes Educator with expertise in cognitive behavioural therapy, motivational interviewing, and diabetes management, met one-on-one each week with study participants using web-conferencing or telephone. Fixed, core elements of the TECC-D intervention included the virtual delivery of the program, the utilization of a health coach to administer the program, weekly touchpoints with participants, and weekly meetings of a virtual interprofessional care team that supported and supplemented the expertise of the health coach.

Health coaching was used as a core component due to its strong evidence of effectiveness compared to standard counselling on promoting treatment adherence and health behaviour change, while reducing healthcare utilization and cost [22,23,26]. Health coaching is defined as the practice of health education and promotion of behaviour change to enhance a person’s well-being and facilitate the achievement of individual health-related goals [27]. Health coaching uses a process of continuous feedback and improvement to provide counselling on goal-setting, negotiate action plans [28], address barriers, and monitor subsequent progress [29,30]. The evidence indicates that health coaching, which comprises ‘low-touch, high-frequency’ contact, supports individuals by enhancing self-efficacy, reducing barriers (real or perceived), improving problem-solving skills, supporting patient-driven goals, and offering tailored feedback and support [24].

**Figure 1:**
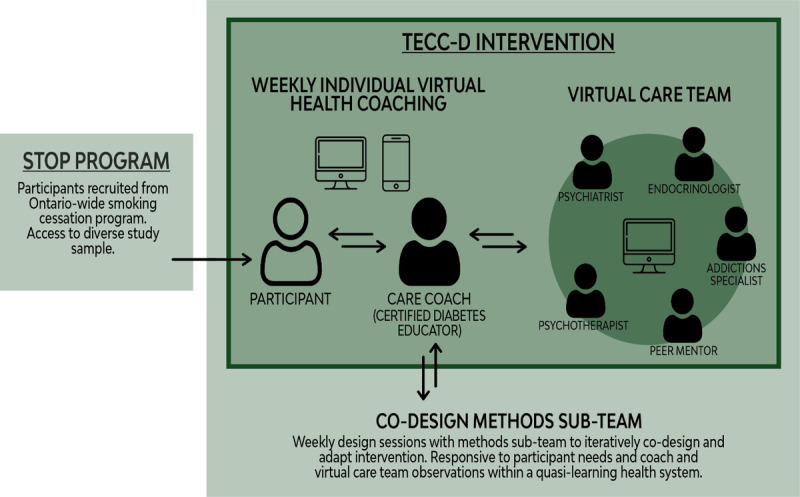
TECC-D Intervention.

Each week the health coach met with an established virtual care team to discuss participant needs, goals, and next steps. Membership of the virtual care team included a peer mentor, addictions specialist, endocrinologist, psychiatrist, and psychotherapist. These meetings were regularly scheduled and, depending on participants need, discussion varied. See Figure 1 for an overview of the TECC-D intervention and supportive processes.

### Outcomes

Primary outcomes (quantitative) included feasibility (i.e., recruitment rate, retention rate) and acceptability (i.e., experience and satisfaction). Secondary outcomes included participant engagement and a process evaluation of the delivery of the intervention (i.e., number of participants enrolled, number of sessions attended, duration of sessions, mode of interaction, and the strategies used by the health coach). Exploratory outcomes included validated physical and mental health tools and questionnaires selected by the study team including patient partners (see Supplemental Table 1 for a full list of questionnaires and tools). To understand the feasibility and acceptability of the TECC-D intervention, study process data included recruitment rate (i.e., the number of enrolled participants divided by the total number of eligible participants), retention rate (i.e., the number of enrolled participants who completed the intervention), drop-out rate (i.e., the number of enrolled participants who did not complete a one-on-one with the health coach), as well as intervention intensity (i.e., number of health coach visits while in the intervention) and mode of interaction (e.g., phone, web-conferencing, or a combination of both).

At baseline, study participants completed a mandatory set of assessments. This set included a demographic and physical health questionnaire (e.g., medication use, stress related to the COVID-19 pandemic); the Diabetes Self-Management Questionnaire [31] to assess diabetes-specific self-care activities associated with glycemic control; the Diabetes Distress Scale [32] to evaluate emotional burden, regimen, interpersonal, and physician-related distress; the EuroQol-5 [33] to assess quality of life; the History of Smoking Index [34] to measure current tobacco use; and the Global Appraisal of Individual Needs Short Screener [35] to measure psychological and behavioural problems. To reduce participant burden, and aligned with feedback received from patient partners, a second set of questionnaires were optional for study participants. These included tools, for example, to measure behavioural change readiness, alcohol use, as well as symptoms of anxiety or depression. Participants could choose what tools, if any, they completed within this second set of questionnaires which were offered at weeks 4, 8, and post-intervention at week 12.

As part of the qualitative design, semi-structured interviews were completed with participants following the completion of the intervention and with virtual care team members at the conclusion of the study. Interviews were completed by phone or web-conferencing, were audio recorded and transcribed verbatim. Participants were asked to reflect on their overall health, their motivation for becoming involved in the TECC-D intervention, what they thought about the intervention, the ways that they felt the intervention contributed to their health, and what they thought could be improved. Virtual care team members, including the health coach, were asked about their involvement in the study, their thoughts on the intervention components and impact, and the future applications of the TECC-D model.

### Data Analysis

A full protocol of the research methods and intervention has been published elsewhere [36]. Briefly, to determine the feasibility and acceptability of the TECC-D program, descriptive statistics were completed to characterize the study sample and describe study process data. Analysis of exploratory outcomes included descriptive statistics with an examination of trends in change. To understand the experience and acceptability of the intervention, interview data were recorded and transcribed, with close coding using the language of the participants through inductive descriptive analysis. Consistent with the chosen approach, a single coder (CW) coded all textual data, identifying any patterns. As data were analyzed concurrently with data collection, as patterns were identified, the study team was engaged in discussion to assist with data interpretation. Study findings, including quantitative and qualitative data are described separately as appropriate, and mixed using a joint display. This joint display integrates the quantitative and qualitative data and help to explain study findings more fulsomely. This study was approved by the Centre for Addiction and Mental Health’s research ethics board in January 2021 (REB:104-2020).

## Results

A total of 31 participants were recruited to participate in this study and completed the intervention between May 2021 and March 2022. Sociodemographic and health characteristic data are presented in Table 1.

**Table 1:** Participant Sociodemographic and Health Characteristics.


PARTICIPANT SOCIODEMOGRAPHIC CHARACTERISTICS (n = 31)		

**Gender**	*n*	Proportion (%)

Woman	15	48.39

Man	16	51.61

**Age**		

Median Age (Range)	61 (37–74)	

	*n*	Proportion (%)

35–45 years	3	9.68

45–55 years	9	29.03

55–65 years	14	45.16

>65 years	5	16.13

**Ethnicity (not mutually exclusive)**	*n*	Proportion (%)

Asian, South-East (i.e., Malaysia, Philippines, Vietnam)	1	3.23

Asian, South (i.e., India, Pakistan, Sri Lanka)	1	3.23

Black, North American (i.e., Canada, America)	1	3.23

First Nations, Inuit, Metis	2	6.45

White, European or North American	23	74.19

Mixed Heritage	2	6.45

Do Not Know	1	3.23

**Marital Status**	*n*	Proportion (%)

Single, Never Married	4	12.90

Married or Domestic Partnership	11	35.48

Widowed	1	3.23

Separated or Divorced	14	45.16

Prefer Not to Answer	1	3.23

**Education Level**	*n*	Proportion (%)

Some High School	5	16.13

High School Diploma	5	16.13

Some College or University	7	22.58

College Diploma or University Degree	14	45.16

**Employment Status**	*n*	Proportion (%)

Currently Working	4	12.90

Not Currently Working	24	77.42

Permanently Unable to Work	3	9.68

**Household Income**	*n*	Proportion (%)

<$10,000	2	6.45

$10,000–$40,000	13	41.94

$40,001–$80,000	9	29.03

$80,001–$100,000	2	6.45

>$100,000	1	3.23

Don’t Know / Prefer Not to Answer	4	12.90

**Participant Health Characteristics (n = 31)**		

**Years with Type-2 Diabetes**		

Median Years (Range)	6.5(1–27)	

**Current Medication Use (Any)**	*n*	Proportion (%)

Yes	30	96.77

No	0	0

Prefer Not to Answer	1	3.23

**Current Insulin Use**	*n*	Proportion (%)

Yes	6	19.35

No	24	77.42

Prefer Not to Answer	1	3.23

**Financial Concern about Diabetes Care**	*n*	Proportion (%)

Yes, a Current Concern	11	35.48

Yes, a Past Concern but Not Now	8	25.81

No, Not a Concern	12	38.71


### Feasibility and Participant Engagement

Recruitment through the STOP program yielded a recruitment rate of 24% (Table 2) with five participants (16%) who consented but did not attend any one-on-one health coaching session. On average, participants were in the intervention for seven weeks, attended six coaching sessions, that lasted 35 minutes. The most common mode of interaction for health coaching was telephone (35%), or a combination of telephone and web-conferencing (23%), with only seven participants choosing to use web-conferencing exclusively (23%).

**Table 2:** Participant Engagement and Intervention Delivery.


MEASURE (n = 31)		

**Participant Recruitment and Engagement**		

**Method of Recruitment**	*n*	Proportion (%)

STOP Program	31	100

**Recruitment Rate**	31/130	23.85%

**Retention Rate**		

Median Number of Weeks BetweenFirst and Last Interaction (Range)	8(0–11.86)	

**Drop-Out Rate**	*n*	Proportion (%)

Did Not Attend One-on-One	5	16.12

**Intervention Delivery**		

**Intensity**		

Median Number of Interactions (Range)	7(0–10)	

**Time Spent Per Interaction**		

Median Time for All Visits in Minutes (Range)	35(6–90)	

**Mode of Interaction**	*n*	Proportion (%)

Telephone only	11	35.48

WebEx only	7	22.58

Combination (Telephone and WebEx)	9	29.03


### Exploratory Outcomes

All participants completed the mandatory baseline questionnaires and tools. However, only 51% of participants chose to complete the 4-week assessments, and 68% chose to complete the 8-week and 12-week assessments. See Supplemental Table 2 for all exploratory findings.

### Experience and Satisfaction

11 participants and four virtual care team members, including one patient partner and the health coach, completed one-on-one interviews. Completed between November 2021 and June 2022, these interviews ranged in duration (28–89 minutes). For each of the TECC-D core components, participant, and virtual care team reflections, including those from the health coach, are offered.

### Virtual delivery

Participants and virtual care team members described the virtual delivery of the program as practical and effective. Participants described value in not having to manage the challenges of in-person appointments for routine care needs.

*“There’s some days where I have no reason to go into town. So, if it’s virtual, I can just sit on my couch comfortably with my cup of coffee. Virtually is easier. Whereas there are days where I don’t feel well, and if I have to go in person, well I don’t. Like, there have been times where I had an appointment at the doctor’s office and I am not feeling well, or my sugars are low or whatever, and I’ve cancelled, or rescheduled because I just didn’t feel it going.” – Participant 21*

Participants appreciated receiving care virtually and found that it saved them money and time (e.g., travel, parking). The health coach experienced the virtual communication with participants and virtual care team members to be efficient.

### Health coach

Participants described having a health coach as “over and above” what their current health care experience can provide. The expertise of the health coach, both in diabetes care and in mental health care, contributed to greater knowledge and skill acquisition for participants, contributed to an enhanced ability to self-manage, and allowed them to set and achieve goals.

*“I think that having the virtual care team benefited me as a provider because it helped to extend my knowledge and insight so that I could be a better coach, or I could help elicit or articulate better what the participant was hoping to achieve and coach them through some of that behaviour change with more insight.” – Health Coach*

For many participants, meeting with the health coach was quite different than the traditional diabetes care that they had received in the past. For example, one participant described the one-on-one nature of the visits as a significant difference for them:

*“The one-on-one, yeah, that was the biggest difference. All the other programs I’ve done for diabetes have been groups, and more, ‘You’re the student, I’m the teacher’ and all in one day. No follow-up. No accountability. Just, here is a bunch of information and do with it what you will, I guess.” – Participant 11*

### Weekly visits

Interview participants described the daily struggles that they experienced related to their diabetes. These challenges included taking daily medications, meal planning, and scheduled physical activity which contributed to perceived stigma and feelings of shame and anger. For one participant, the burden of managing diabetes was described to be a daily struggle:

*“For the longest time I would avoid restaurants. I was angry about [having diabetes]. There’s so much I want to do but to have to stop everything I’m doing and to check my sugars and to make sure that I’m eating right. Like, I wanted to go for walks, enjoy myself. I got angry for many years because I can’t just go for a walk right now or I can’t just go for a hike because I have to remember to bring a snack and, you know, I guess I couldn’t be spontaneous, you know what I mean? I’ve come to terms with the fact that, you know, this is what it is, and I have to live around it.” – Participant 21*

While the weekly visits from the health coach were described as helpful, participants described an opportunity for further flexibility regarding the frequency of the program visits as well as the overall duration of the program. For example, while many participants were satisfied with eight visits over eight weeks, several participants felt that they would have benefited from a longer intervention period with weekly visits, others described wanting fewer touchpoints but over a sustained period of time. This was reflective of individual needs and context.

In addition, both participants and the health coach identified an opportunity for a more gradual discharge from the program. This could include scheduled visits several weeks into the future following the completion of the program as a means of checking in:

*“I would’ve thought another couple weeks. It felt like, maybe, even during the last visit if we had scheduled another visit, maybe a few weeks later, just to like, well, ‘How are you doing so far?’ and then I could have shared what I’ve done. That sort of thing?” – Participant 11*

### Virtual care team

Virtual care was described as a positive for both the participant and the whole virtual care team. This included increased knowledge and skill (e.g., smoking cessation, learning from lived experience) reflective of the collaborative professional expertise that the team possessed.

For the health coach, the virtual care team supported their professional development, including their knowledge and ability to support individuals with mental health concerns.

*“I looked at the virtual care team as sort of like coaching for the coach, right? I learned a lot. I learned a lot from a mental health perspective. That helped me grow as a professional, for sure. So if anything, the virtual care team was for me, not the participant, if you will.” – Health Coach*.

While this support of the virtual care team was generally regarded as positive, there were also challenges. For example, because the virtual care team never met with the participant, the health coach described initial hesitancy with passing along information or sharing new knowledge. However, from the participant perspective, only meeting with the coach was regarded as positive as it allowed them to have one contact person, provided a sense of accountability, and permitted relationship building.

### Integrated, collaborative care model

Recognized as interconnected, participants appreciated the clinical integration of physical and mental health care in the program. This was described as “missing from diabetes care” and, for one participant, acknowledged that they were “more than just the sum of my parts”. This integration offered an opportunity for the health coach to speak to participants about the significant psychosocial aspects and challenges of living with diabetes, including the daily struggle described by participants.

While the impact of this clinical integration was described by both participants and virtual care team members, one of the identified gaps was the need for more robust linkages between this program and other health services. For example, while the health coach could advocate for self-referrals to community organizations or programs, they were unable to directly refer to a specific person or program. Further, virtual care team members recognized the need for connection to primary care or further integration at the professional and organization level.

*“I think one of the main challenges was that we haven’t ironed out completely was how we link this person back to their family doctor? How do we send information? And there was one point that I prepared a note for one of the participants to send a letter to their family doctor saying, “This came up. These are some of the suggestions we have.”– Virtual Care Team Member*

### Intervention Delivery

There were several strategies and recommendations posed by the health coach during one-on-one visits. Both quantitative and qualitative data, including quotes from participants, virtual care team members, and chart data, is organized in a joint display (see Table 3). While all strategies and recommendations were applied by the health coach during the one-on-one visits, certain strategies, including psychosocial support, behavioural change, and self-management education were most used.

**Table 3:** Joint Display of TECC-D Strategies and Recommendations.


TECC-D IMPLEMENTATION		DESCRIPTION/QUOTE/CHART DATA

**Strategy and Recommendations**		

**Diabetes Self-Management Education/Support**	n = 25	Participants described receiving broad information and resources regarding diabetes self-management and support. This included information about diet, education specific to interpreting lab values, identifying patterns and assisting with pattern management, exercise, medications, and linking pathophysiology of diabetes to treatment to enhance goal development. *“She gave me options, like, at the diabetes center I attend, they gave me one particular way to eat during the day. [Health Coach] gave me more options. The fact that I could have a regular appointment with someone to discuss this was helpful.” – Participant 21*

Number of Times Strategy Used	82	

**Glucose Monitoring**	n = 14	

Number of Times Recommendation Used	21	

**Diabetes Medication Adjustment**	n = 2	

Number of Times Recommendation Used	2	

**Behavioural Change**	n = 23	Topics such as motivation and habits were discussed as participants identified personal health goals. Supported by Diabetes Canada recommendations and guidelines, [37] and using a tiny habit approach, the health coach shared potential options for change and assisted participants in articulating and refining their goals. *“It never felt like we were doing anything really structured. It was like having a chat with a friend, but that friend had all this knowledge and this team of specialists to support her. It didn’t feel like she was following an itinerary or that she had specific steps to follow. It was organic and specific to me, and I think that’s why I was able to make some big changes for both my physical and mental health – changes that I have needed to make for many, many years.” – Participant 31*

Number of Times Strategy Used	87	

**Diabetes Medication Adherence**	n = 10	

Number of Times Recommendation Used	13	

**Dietary Modification**	n = 21	

Number of Times Recommendation Used	39	

**Exercise Modification**	n = 11	

Number of Times Recommendation Used	26	

**Case Management**	n = 25	Based on participant need, the health coach would research and connect participants to both diabetes and non-diabetes-specific resources. For example, this included diabetes education resources or addiction programs. *“Like, I go to these diabetes classes, right? But we don’t talk about how diabetes is different for each of us. Like, I have friends with diabetes, and they don’t worry about food at all. I worry a lot.” – Participant 23*

Number of Times Strategy Used	76	

**System Navigation**	n = 17	

Number of Times Recommendation Used	31	

**Psychosocial Support**	n = 24	Comprehensive psychosocial support was provided by the health coach. This included the use of motivational interviewing to build rapport and promote a supportive environment where participants felt they could identify challenges and struggles, work through them, and celebrate success.

Number of Times Strategy Used	111	

**Other**	n = 9	Participants identified broad goals and needs during study visits. For example, and in addition to specific diabetes and health needs, this included support with smoking cessation, alcohol use, and past trauma. The health coach encouraged self-referrals to community supports specific to participant need. *“We started focusing on that and where I could get help with my alcohol use. I’m in a group now. I go every Thursday.”– Participant 10*

Number of Times Strategy Used	12	

**External Referral**	n = 9	

Number of Times Recommendation Used	11	


## Discussion

For individuals living with T2D and mental health concerns, health care is typically delivered across different care providers, and in different care settings. Considering the demonstrated interconnectedness of health risk behaviours that contribute to, challenge, and develop because of T2D alongside the high prevalence of co-occurring diabetes and mental health concerns, there is an opportunity to address this system and clinical disconnectedness. The TECC-D program was both co-designed and redesigned to address this complexity and respond to a need for integrated care at a clinical and professional level. Findings from this mixed methods feasibility study demonstrate that the TECC-D model is both a feasible and scalable care solution for those living with co-occurring T2D and mental health concerns. Recruitment from a large province-wide registry of individuals who are current or past cigarette smokers offered a direct and effective mechanism of including participants from a typically hard-to-reach population of those living with chronic illness and co-morbid mental health concerns who are at a high risk of health complications. The TECC-D program itself, co-designed iteratively and supported by innovative agile and real-time adaptations to the model, led to good uptake of the program and may support dissemination and scale-up.

Globally, there are a growing number of integrated diabetes and mental health programs supported by technology [38]. Like the TECC-D model, many of these models focus on promoting wellness, preventing complications, and enhancing self-management and treatment for those living with diabetes and co-occurring mental health challenges [38]. However, unique to the TECC-D model is the embrace of existing and widely-available technology to enable this connection and care. Driven by the relationships established by the health coach, and leveraging assets that exist in the current healthcare system, this feasibility study has demonstrated that slick technology or new applications are not needed to enhance and improve access to integrated healthcare. While only a few other technology supported diabetes and mental health programs have used technology to enable better access to healthcare [39,40], the implications for this approach are far reaching, particularly for equity deserving groups.

This study has several strengths. First, the broad inclusion criteria enabled the evaluation of the program in a diverse sample of participants with varying demographic and health characteristics. Second, by leveraging existing assets of the study team (i.e., the TECC model, evidence in support of health coaching, and the recruitment sample), the co-designed program is pragmatic, and responsive to the real-world health care context. Third, and reflective of the need to learn in real-time, the use of iterative co-design, including patient partners and the study team, was innovative and aligned to broader use of learning health systems.

There were many learnings that came from this work. Limitations of this feasibility study include the small sample size which did not permit a full analysis of exploratory outcomes. This extends to the ways that acceptability of the program was tested. In future work, there is an opportunity to additionally consider concepts such as the burden of the intervention [41] on both participants and healthcare professionals. Further, it is unknown whether a selection bias may have influenced study findings as evidenced by one participant’s reflections, “I suspect that the only people who are going to participate in a program like this are the people who really want to help themselves.” Responding to this potential limitation, future evaluation of the program should include pragmatic methods in real-world conditions and comparisons between those who do and do not choose to participate. In using a province-wide registry of individuals who smoke for recruitment, the study population included an oversampling of those adults with diabetes who smoke cigarettes. While this may also be a strength given the known linkages between smoking, macrovascular diabetes complications, diabetes, and other mental health concerns, there is a need for future research to test this program in individuals who do not smoke.

Several study limitations were introduced as part of the extensive co-design work that was completed. For example, while patient partners chose the 8-week duration of the intervention, there is evidence that indicates that a longer timeframe or longer-term follow-up for these types of interventions is needed [42,43]. Based on this evidence and aligned to study findings, at minimum, a follow-up period after the initial 8 weeks should be tested in future iterations of this program. Further, challenges with data collection, including when tools were completed, and the balance between collecting information without further stigmatizing those living with highly stigmatized health conditions, limited the data available to analyze. As a result, there is future work to be done to identify an acceptable screening tool for mental health status at baseline, for example, that does not introduce unnecessary stigma or limit engagement in the program. Lastly, the missing linkage between the health coach and participant’s existing primary care team challenged how information could be shared and ultimately, how care could be co-managed.

## Conclusion

The TECC-D model is a feasible and scalable virtual care solution that empowers individuals living with T2D and mental health concerns to take an active role in improving their physical and mental health. There is an opportunity and need to further test this model for its effectiveness in improving patient outcomes.

## Additional File

The additional file for this article can be found as follows:

10.5334/ijic.7608.s1 Supplemental File 1. Supplemental Tables 1 and 2.
