# Supplementary material for: Technology-Enabled Collaborative Care for Type-2 Diabetes and Mental Health (TECC-D): Findings From a Mixed Methods Feasibility Trial of a Responsive Co-Designed Virtual Health Coaching Intervention
Source: Int J Integr Care. 2024 Feb 16;24(1):12. doi: 10.5334/ijic.7608 (PMC10870944; doi:10.5334/ijic.7608)
Supplement: Supplemental File 1. — Supplemental Tables 1 and 2. [file ijic-24-1-7608-s1.pdf]

Supplemental Table 1. Study tools and questionnaires.

Questionnaire or Tool	Brief Description of Measure	Timepoints Available to Complete
Body mass index	Self-report	Baseline, 4, 8, 12 week
Blood pressure	Self-report	Baseline, 4, 8, 12 week
HbA1c	Self-report	Baseline, 4, 8, 12 week
Diabetes Self-Management Questionnaire	Diabetes self-care activities associated with glycemic control	Baseline, 4, 8, 12 week
Diabetes Distress Scale	Diabetes emotional burden, regimen, interpersonal, and physician-related distress	Baseline, 8-week
EuroQol- 5 Dimension	Quality of life	Baseline, 4, 8, 12 week
History of Smoking Index	Tobacco use	Baseline, 4, 8, 12 week
Global Appraisal of Individual Needs Short Screener	Psychological and behavioural health symptoms	Baseline
Readiness Ruler	Assess motivation and confidence regarding health behaviours (e.g., physical activity, nutrition, smoking, alcohol, stress, sleep)	Baseline, 4, 8, 12 week
Alcohol Use Disorders Identification Test	Alcohol use	Baseline, 4, 8, 12 week
Mediterranean Diet Adherence Screener	Measure adherence to the Mediterranean diet	Baseline, 4, 8, 12 week
International Physical Activity Questionnaire – Short Form	Physical activity	Baseline, 4, 8, 12 week
Brief Pain Inventory	Pain symptoms	Baseline
Generalized Anxiety Disorder Scale	Anxiety symptoms	Baseline, 4, 8, 12 week
Patient Health Questionnaire	Depressive symptoms	Baseline, 4, 8, 12 week
Perceived Stress Scale	Stress symptoms	Baseline, 8-week
Diabetes Self-Management Technology Questionnaire	Use of technology in diabetes management	Baseline

Supplemental Table 2. Completion of Study Assessments and Exploratory Outcomes

**Completion of Study Assessments**	**Number Completed**	**Proportion (%)**
Baseline Assessment	31	100
4-Week Assessment	16	51.61
8-Week Assessment	21	67.74
12-Week Assessment	21	67.74
**Exploratory Outcome**	**Pre-Intervention**	**Post-Intervention**
**Health Behaviour**		
**International Physical Activity Questionnaire (IPAQ)**	n=5	n=2
Mean Number of Minutes of Vigorous Activity per Active Day (SD)	55(40.31)	80(56.57)
Median Number of Minutes of Vigorous Activity per Active Day (Range)	50(15-120)	80(40-120)
	n=5	n=4
Mean Number of Minutes of Moderate Activity per Active Day (SD)	48(16.43)	35(17.32)
Median Number of Minutes of Moderate Activity per Active Day (Range)	60(30-60)	30(20-60)
	n=10	n=7
Mean Number of Minutes Walking per Active Day (SD)	51.11(42.92)	25(7.75)
Median Number of Minutes Walking per Active Day (Range)	30(15-120)	30(15-30)
	n=11	n=7
Mean Number of Minutes Spent Sitting each Weekday (SD)	420(250.67)	402.86(207.02)
Median Number of Minutes Spent Sitting each Weekday (Range)	390(60-900)	360(180-720)
**Mediterranean Diet Adherence Screener (MEDAS)**	n=13	n=11
Mean MEDAS Score (SD)	5.08(2.56)	4.72(1.79)
Median MEDAS Score (Range)	6(1-9)	5(1-8)
**Readiness to Change Rulers**		
	n=17	n=11
Mean Confidence to Change Diet (SD)	7.94(1.71)	7.45(1.91)
Median Confidence to Change Diet (Range)	7(5-10)	8(4-10)
	n=17	n=11
Mean Importance to Change Diet (SD)	8.00(2.12)	8.30(1.34)
Median Importance to Change Diet (Range)	8.5(3-10)	8.5(6-10)
	n=17	n=11
Mean Confidence to Change Physical Activity (SD)	7.18(1.84)	6.63(2.11)
Median Confidence to Change Physical Activity (Range)	7(3-10)	6(3-10)

	n=17	n=11
Mean Importance to Change Physical Activity (SD)	8.00(2.13)	7.70(2.31)
Median Importance to Change Physical Activity (Range)	8.5(3-10)	8(2-10)
	n=11	n=9
Mean Confidence to Change Smoking Intake (SD)	5.91(3.02)	6.67(2.92)
Median Confidence to Change Smoking Intake (Range)	6(1-10)	6(1-10)
	n=11	n=9
Mean Importance to Change Smoking Intake (SD)	7.40(3.20)	8.00(2.96)
Median Importance to Change Smoking Intake (Range)	8.5(1-10)	9(1-10)
	n=10	n=6
Mean Confidence to Change Alcohol Consumption (SD)	7.50(2.88)	8.50(2.74)
Median Confidence to Change Alcohol Consumption (Range)	8.50(1-10)	9.50(3-10)
	n=10	n=6
Mean Importance to Change Alcohol Consumption (SD)	5.83(3.86)	9.00(1.73)
Median Importance to Change Alcohol Consumption (Range)	7(1-10)	10(6-10)
	n=16	n=10
Mean Confidence to Change Stress (SD)	7.69(2.39)	7.10(1.45)
Median Confidence to Change Stress (Range)	8(1-10)	7(5-9)
	n=16	n=10
Mean Importance to Change Stress (SD)	6.06(2.74)	7.22(2.73)
Median Importance to Change Stress (Range)	6.5(1-10)	8(1-10)
	n=15	n=9
Mean Confidence to Change Sleep (SD)	7.00(2.90)	6.67(2.06)
Median Confidence to Change Sleep (Range)	7(1-10)	6(5-10)
	n=15	n=9
Mean Importance to Change Sleep (SD)	6.78(3.58)	8(1.77)
Median Importance to Change Sleep (Range)	7.5(1-10)	8(5-10)
**Substance Use**		
**Global Appraisal of Individual Needs Short Screener (GAIN-SS)**	n=27	
Mean Internalized Disorder Score (SD)	2.33(1.52)	
Median Internalized Disorder Score (Range)	2(0-5)	
Mean Externalized Disorder Score (SD)	1.37(1.42)	
Median Externalized Disorder Score (Range)	1(0-4)	
Mean Substance Disorder Screen (SD)	0.70(0.82)	

Median Substance Disorder Screen (Range)	0(0-2)			
Mean Crime and Violence Score (SD)	0.15(0.36)			
Median Crime and Violence Screen (Range)	0(0-1)			
**Alcohol Use Disorders Identification Test (AUDIT)**	n=3		n=3	
Mean AUDIT-3 Score (SD)	8.67(1.53)		6.67(5.51)	
Median AUDIT-3 Score (Range)	9(7-10)		7(1-12)	
Mean AUDIT Total Score (SD)	15.00(1.73)		13.33(11.59)	
Median AUDIT Total Score (Range)	14(14-17)		15(1-24)	
**Heaviness of Smoking Index 2-item (HSI-2)**	n=20		n=16	
Mean HSI-2 Score (SD)	2.9(1.55)		2.93(1.57)	
Median HSI-2 Score (Range)	3(0-5)		3(0-5)	
**Mental Health**				
**Patient Health Questionnaire 2-item (PHQ-2)**	n=4		n=2	
Mean PHQ-2 Score (SD)	4(1.41)		3.5(0.5)	
Median PHQ-2 Score (Range)	3.50(3-6)		3.5(3-4)	
**Patient Health Questionnaire 9-item (PHQ-9)**	n=4		n=2	
Mean PHQ-9 Score (SD)	16.25(9.43)		12(8)	
Median PHQ-9 Score (Range)	17(4-27)		12(4-20)	
**Generalized Anxiety Disorder 2-item (GAD-2)**	n=8		n=6	
Mean GAD-2 Score (SD)	2.50(1.41)		3.00(2.00)	
Median GAD-2 Score (Range)	2(2-6)		2(1-6)	
**Generalized Anxiety Disorder 7-item (GAD-7)**	n=8		n=6	
Mean GAD-7 Score (SD)	7.88(5.68)		9.17(6.27)	
Median GAD-7 Score (Range)	6.5(2-21)		8.5(1-19)	
**Perceived Stress Scale (PSS)**	n=9		n=8	
Mean PSS Score (SD)	16.33(10.48)		22.13(5.36)	
Median PSS Score (Range)	17(2-36)		20.5(16-33)	
**Diabetes Distress Scale (DDS)**	n=29		n=21	
Mean DDS Score (SD)	2.03(0.75)		1.89(0.61)	
Median DDS Score (Range)	1.71(1.05-4.06)		1.88(1.12-3.12)	
**EuroQoL 5-item (EQ-5D)**				
Mobility	n	Proportion (%)	n	Proportion (%)
No Problems	7	25.93	5	23.81
Slight Problems	10	37.04	8	38.10
Moderate Problems	5	18.52	6	28.57
Severe Problems	4	14.81	2	9.52
Unable to Walk	1	3.70	0	0

Missing	4		10	
Self-Care	n	Proportion (%)	n	Proportion (%)
No Problems	20	76.92	11	55.00
Slight Problems	5	19.23	7	35.00
Moderate Problems	1	3.85	2	10.00
Severe Problems	0	0	0	0
Unable to Wash or Dress Self	0	0	0	0
Missing	5		11	
Usual Activities	n	Proportion (%)	n	Proportion (%)
No Problems	8	29.63	5	23.81
Slight Problems	12	44.44	11	52.38
Moderate Problems	6	22.22	4	19.05
Severe Problems	1	3.70	1	4.76
Unable to Do Usual Activities	0	0	0	0
Missing	4		10	
Pain or Discomfort	n	Proportion (%)	n	Proportion (%)
No Pain or Discomfort	3	11.11	2	9.52
Slight Pain or Discomfort	8	29.63	10	47.62
Moderate Pain or Discomfort	11	40.74	6	28.57
Severe Pain or Discomfort	4	14.81	2	9.52
Extreme Pain or Discomfort	1	3.70	1	4.76
Missing	4		10	
Anxiety or Depression	n	Proportion (%)	n	Proportion (%)
No Anxiety or Depression	7	25.93	6	30.00
Slight Anxiety or Depression	11	40.74	7	35.00
Moderate Anxiety or Depression	5	18.52	5	25.00
Severe Anxiety or Depression	2	7.41	1	5.00
Extreme Anxiety or Depression	2	7.41	1	5.00
Missing	5		12	
	n=27		n=19	
Mean Quality of Life EQ-5D Score (SD)	64.89(20.78)		66.42(20.47)	
Median Quality of Life EQ-5D (Range)	70(20-94)		70(30-100)	
**Physical Health**				
**Body Mass Index (BMI) Self-Report**	n=28		n=21	
Mean BMI (SD)	33.31(7.97)		33.88(7.09)	
Median BMI (Range)	33.55(21.10-53.30)		35.10(21.10-50.00)	
**Blood Pressure Self-Report**	n=14		n=10	
Mean Systolic Blood Pressure (SD)	124.21(8.75)		127.40(11.96)	

Mean Diastolic Blood Pressure (SD)	78.07(7.28)	77.30(8.04)
Median Systolic Blood Pressure (Range)	128(103-135)	128.5(105-150)
Median Diastolic Blood Pressure (Range)	77(69-90)	75.5(68-90)
**HbA1C Level**	n=21	n=8
Mean HbA1C (SD)	7.37(1.55)	7.37(1.21)
Median HbA1C (Range)	6.90(5.90-12.00)	6.80(6.50-10.20)
**Diabetes Self-Management Technology Questionnaire (DSMT-Q)**	n=29	
Mean Total DSMT-Q Score	5.59(1.52)	
Median Total DSMT-Q Score	5.41(2.29-8.33)	
Mean Glucose Management Score (SD)	6.41(2.04)	
Median Glucose Management Score (Range)	6.00(3.33-10.00)	
Mean Dietary Control Score (SD)	4.64(1.79)	
Median Dietary Control Score (Range)	5.00(1.66-8.33)	
Mean Physical Activity Score (SD)	5.75(2.14)	
Median Physical Activity Score (Range)	5.56(1.11-10.00)	
Mean Health Care Use Score (SD)	7.81(2.42)	
Median Health Care Use Score (Range)	8.89(2.22-10.00)	
**Brief Pain Inventory (BPI)**	n=9	
Mean Total Pain Impact (SD)	3.75(0.93)	
Median Total Pain Impact (Range)	4(1.7-4.5)	
Mean General Activity Impact (SD)	4.2(3.67)	
Median General Activity Impact (Range)	5(0-10)	
Mean Mood Impact (SD)	4(3.84)	
Median Mood Impact (Range)	4(0-10)	
Mean Walking Ability Impact (SD)	3.44(3.50)	
Median Walking Ability Impact (Range)	4(0-8)	
Mean Work Impact (SD)	4.22(3.83)	
Median Work Impact (Range)	4(0-10)	
Mean Relationships With Other People Impact (SD)	1.77(3.35)	
Median Relationships With Other People Impact (Range)	0(0-10)	
Mean Sleep Impact (SD)	4.55(3.64)	
Median Sleep Impact (Range)	5(0-9)	
Mean Enjoyment of Life Impact (SD)	4(3.46)	
Median Enjoyment of Life Impact (Range)	5(0-10)	
